# Analysis of therapeutic targets and prognostic biomarkers of CXC chemokines in cervical cancer microenvironment

**DOI:** 10.1186/s12935-021-02101-9

**Published:** 2021-07-28

**Authors:** Weina Kong, Gang Zhao, Haixia Chen, Weina Wang, Xiaoqian Shang, Qiannan Sun, Fan Guo, Xiumin Ma

**Affiliations:** 1 State Key Laboratory of Pathogenesis, Prevention and Treatment of High Incidence Diseases in Central Asia, Department of Clinical Laboratory Center, Tumor Hospital Affiliated to Xinjiang Medical University, No 789 Suzhou Road, Ürümqi, China; 2 Department of Blood Transfusion, Affiliated Traditional Chinese Medicine Hospital of Xinjiang Medical University, Ürümqi, China; 3 Department of Pathology, Tumor Hospital Affiliated to Xinjiang Medical University, Ürümqi, China

**Keywords:** Cervical cancer, CXC chemokine, Quantitative real-time PCR, Immunohistochemistry, Prognosis, Tumor microenvironment

## Abstract

**Background:**

The tumor microenvironment (TME) has received an increasing amount of attention. CXC chemokines can regulate immune cell transport and tumor cell activity to exert anti-tumor immunity. However, studies on the expression and prognosis of CXC chemokines in cervical cancer (CC) are more limited.

**Methods:**

The study investigated the role of CXC chemokines in TME of CC by using public databases. Moreover, quantitative real-time PCR (qRT-PCR) and immunohistochemistry (IHC) of CXC chemokines were performed to further verify.

**Results:**

The transcriptional levels of CXCL1/3/5/6/8/9/10/11/13/16/17 in CC tissues were significantly elevated while the transcriptional levels of CXCL12/14 were significantly reduced. We reached a consistent conclusion that the expression of CXCL9/10/11/13 was verified by quantitative real-time PCR and immunohistochemistry. Moreover, CC patients with low transcriptional levels of CXCL1/2/3/4/5/8 were significantly associated with longer overall survival (OS). The CCL family was related to CXC chemokines neighboring alteration. RELA, NFKB1, LCK and PAK2 were the key transcription factors and kinase targets of CXC chemokines, respectively. We also found there were significant correlations between the expression of CXCL9/10/11 and the infiltration of immune cells (CD8+ T cell, CD4+ T cell, neutrophils and dendritic cells).

**Conclusions:**

In brief, we conducted a comprehensive analysis of CXC chemokines via clinical data and some online public databases. Our results may provide a new idea for the selection of immunotherapeutic targets and prognostic biomarkers for cervical cancer.

**Supplementary Information:**

The online version contains supplementary material available at 10.1186/s12935-021-02101-9.

## Background

Cervical cancer accounts for approximately 12% of female cancers and is one of the major causes of death among women worldwide [1, 2]. Although the early detection and treatment of CC have improved, there are still about 604,127 new cases and 341,831 deaths in 2020, according to data from the World Health Organization [3]. The actual situation shows that the majority of patients with cervical cancer are in the advanced stage of the disease, with limited access to appropriate treatment [4]. As a result, the mortality rate is still high, and the median overall survival rate of advanced cervical cancer is only 16.8 months [5]. Nowadays, an increasing number of studies have explored the kinase and immune checkpoint inhibitors in cancer [6]. Moreover, epigenetics, the new regulation of specific genes, has also shown certain importance in the progression of cervical cancer [7]. This provides a new idea for identifying the therapeutic targets and prognostic biomarkers of cervical cancer.

Chemokines are a type of secreted proteins with small molecular weights. Their role in autoimmune diseases, chronic inflammations and tumors has been continuously revealed [8], mainly mediating the migration of immune cells and the development of lymphoid tissues [9]. In recent years, the role of chemokines in the tumor microenvironment has been continuously reported by researchers. As a major subfamily of the chemokines family, the function and mechanism of CXC chemokines have been discovered in tumors [10]. CXC chemokines’ altered expression in cancers dictates immune cell recruitment, angiogenesis, tumorigenesis, cancer cell proliferation and metastasis [11, 12]. Previous studies have shown that there is a correlation between CXC chemokines, tumor microenvironment and tumor immunotherapy [13], which has been confirmed in some cancers [14–16]. This suggests that CXC chemokines may be potential therapeutic targets and prognostic biomarkers of cancer, by modulating tumor progression and immunotherapy efficacy. However, the function of the CXC chemokine family in CC has not been comprehensively described.

CXC chemokine is an important component of TME. Although previous studies have confirmed the expression and role of some members of the CXC chemokine family in cervical cancer, there is still a lack of comprehensive and systematic research. Therefore, it is worth exploring CXC chemokines as therapeutic targets and prognostic markers of cervical cancer. In this study, public databases were used to investigate the mRNA expression, prognosis, and related targets or kinase pathways of the CXC chemokine family in CC. Immunohistochemistry and qRT-PCR further verified the conclusion. Taken together, this study complements the function of CXC chemokines in cervical cancer, suggesting that certain CXC chemokines can be used as potential therapeutic targets and prognostic biomarkers for CC.

## Materials and methods

### Study population

In this study, clinical data and pathological specimens were collected retrospectively, and the patients’ informed consent was obtained before the pathological specimens were collected. Cervical cancer and para-carcinoma tissue samples for qRT-PCR and IHC analysis were taken from patients who underwent surgery from January 1, 2017 to December 31, 2018. We collected frozen tissues of 60 patients to analyze the mRNA levels of CXC chemokines by qRT-PCR and analyzed the protein expression levels of CXC chemokines by IHC staining in the paraffin tissues of 60 patients. This research work had been approved by the Academic Committee of The Third Clinical Medical College of Xinjiang Medical University (affiliated Tumor Hospital) and was carried out under the rules put forward in the Declaration of Helsinki. This study had the relevant informed consent exemption certificate issued by the Academic Committee. As for the public databases, neither ethics committee approval nor patient informed consent was needed for analyzing data.

### Quantitative real-time PCR

Total RNA was isolated from tumor tissues and adjacent tissues using Trizol according to the manufacturer’s instructions. The extracted RNA was converted into cDNA with 5× primescript buffer, prime script RT enzyme mix I, oligo-dT primer and random 6 mers. The qRT-PCR was performed in the BioRad CFX96 Real-Time PCR Detection System machine in the presence of GAPDH, CXCL9, CXCL10, CXCL11, CXCL12 and CXCL13. We verified CXCL9/10/11/13 because these factors were expressed on the intersection of the three common databases including ONCOMINE, GEPIA and UALCAN. The detection of CXCL12 was because it was the only one that tended towards low expression in two databases. The transcription level of target genes was measured and normalized to GAPDH expression. The following primer sequences were used: GAPDH, 5′-GAAGGTGAAGGTCGGAGTC-3′ (forward) and 5′-GAAGATGGTGATGGGATTTC-3′ (reverse); CXCL9, 5′-TGAGAAAGGGTCGCTGTTCC-3′ (forward) and 5′-GGGCTTGGGGCAAATTGTTT-3′ (reverse); CXCL10, 5′-TGCCATTCTGATTTGCTGCC-3′ (forward) and 5′-TGCAGGTACAGCGTACAGTT-3′ (reverse); CXCL11, 5′-CCCTGGGGTAAAAGCAGTGA-3′ (forward) and 5′-TAAGCCTTGCTTGCTTCGAT-3′ (reverse); CXCL12, 5′-AGATGCCCATGCCGATTCTT-3′ (forward) and 5′-AGGGCACAGTTTGGAGTGTT-3′ (reverse); CXCL13, 5′-CGACATCTCTGCTTCTCATGC-3′ (forward) and 5′-ACTGAGCTCTCTTGGACACAT-3′ (reverse).

### Immunohistochemistry

Formalin-fixed paraffin-embedded surgical specimens were used for immunohistochemical study. The sections were dried at 60 °C for 2 h, subsequently were dewaxed in xylene and graded alcohols, were hydrated and washed in phosphate-buffered saline. After antigen repair was treated in a microwave oven (15 min in citrate buffer, pH 6.0), the endogenous peroxidase was inhibited with 3% H_2_O_2_ for 30 min, then the sections were incubated with 10% normal goat serum for 40 min. Primary antibodies composed of rabbit anti-CXCL9 antibody (bs-2551R [Bioss], 1:100), rabbit anti-CXCL10 antibody (bs-1502R [Bioss], 1:150), rabbit anti-CXCL11 antibody (DF9917 [Affinity], 1:150) and rabbit anti-CXCL13 antibody (bs-2553R [Bioss], 1:100) were applied overnight in a moist room at 4°C. Then the tissues were incubated with secondary antibody (37 °C 50 min), stained with diaminobenzidine, and counterstained with hematoxylin. Positive staining was evaluated using computer-aided image analysis and Image J software. The average CXC chemokines infiltration was determined from three randomized fields by two independent pathologists who were unaware of the patients’ pathological and clinical status.

### Transcription-related databases of CXC chemokines in patients of cervical cancer

We used the public databases ONCOMINE (http://www.oncomine.org) [17], GEPIA (http://gepia.cancer-pku.cn/index.html) [18] and UALCAN (http://ualcan.path.uab.edu) [19] that could provide cancer RNA sequence data and clinical data to analyze the differential expression of CXC chemokines in cervical cancer and adjacent cancer or normal tissues by using Student’s *t*-test. The cut-off of the *p*-value was 0.05. In ONCOMINE, the fold change was 2.0, and the gene rank was in the top 10%. In brief, we entered the target genes in the input box of the database, and then searched for them. In addition, we conducted a prognostic study of CXC chemokines in cervical cancer by Kaplan–Meier curve in the GEPIA database.

### cBioPortal

cBioPortal (http://www.cbioportal.org) is an online open-access website, which involves the exploration, visualization, and analysis of multidimensional cancer genomics data [20]. Genetic alterations of CXC chemokines were obtained from cBioPortal based on The Cancer Genome Atlas (TCGA) database. In this study, 293 cervical squamous cell carcinoma samples were analyzed (TCGA, PanCancer Atlas). The *z*-score of mRNA expression (log RNA Seq V2 RSEM) was obtained using the threshold of ± 2.0.

### CXC chemokines-related networks

We studied the gene-related networks and protein–protein interaction (PPI) of CXC chemokines by GeneMANIA (http://www.genemania.org) [21], a website which could provide information on the protein and genetic interactions, pathways and co-expression of submitted genes, and the STRING database (https://string-db.org/) [22], respectively. In our research, we entered the searched species and gene names in the input box of databases. Moreover, on the right side of the GeneMANIA website, we also set up bioinformatics methods such as co-expression, physical interaction, gene enrichment analysis, predictive interaction and pathway, etc.

### Gene Ontology (GO) and Kyoto Encyclopedia of Genes and Genomes (KEGG) analysis

DAVID 6.8 (https://david.ncifcrf.gov/home.jsp), a website that can clarify the biological functions of genes [23], was used to perform GO and KEGG pathway enrichment analysis of CXC chemokines and their neighboring 50 genes. Analysis of GO function mainly included biological process (BP), cell component (CC), molecular function (MF). Then, the “ggplot2” package of R software (4.0.2) was used for visualization. *P*-value < 0.05 was considered to be statistically different.

### Target analysis of kinases and transcription factors

The TRRUST (https://www.grnpedia.org/trrust/) database can provide transcription factor (TF)–target regulatory relationship [24]. The module of LinkInterpreter in the LinkedOmics (http://www.linkedomics.org/) [25] was used to obtain biological insights on the enrichment of kinase targets of CXC chemokines. In the LinkedOmics database, Gene Set Enrichment Analysis (GSEA) was investigated with a minimum number of genes (size) of 3 and a simulation of 500. The results were analyzed by the Spearman correlation test. The *p*-value cut-off was 0.05.

### TIMER database

TIMER (https://cistrome.shinyapps.io/timer/) could provide a systematic assessment of the infiltration of different immune cells and their clinical effects [26]. We conducted the module of correlation in the TIMER database to evaluate the interrelation between immune cell infiltration and CXC chemokines level (or CXCRs) by the purity-corrected partial Spearman method in the tumor microenvironment. Moreover, the correlations between cell markers of CD8+ T cells (CD8A and CD8B), natural killer (NK) cells (KLRK1, KIR2DL4, KIR3DL2, NCR1, and NCR3), T helper 1 (Th1) cells (TBX21, STAT1) and CXCL9–11/CXCR3 were explored.

### Statistical analysis

The data of clinical patients were presented as mean ± SD, the Chi-square test and Wilcoxon rank-sum test was used to compare the data between the tumor group and the para-cancerous group (SPSS 26.0). The qRT-PCR and immunohistochemical data of 60 patients were analyzed using Student’s *t*-test and Mann–Whitney *U* test (Graphpad Prism 8.0). Differences with *p* values < 0.05 were considered statistically significant.

## Results

### The clinicopathological characteristics of the patients

The clinicopathological features of 60 patients were studied. The results are shown in Table 1. There were no differences in ethnicity (*p* = 0.566), tumor size (*p* = 0.756), differentiation (*p* = 1.000), or FIGO stage (*p* = 0.378) between the tumor tissue group and the adjacent tissue group, except for first diagnostic age (*p* = 0.000).

**Table 1:** Clinical characteristics of patients with cervical cancer

Clinical or pathologic feature	*N* (%) or mean (s.d.)		*p* value
	Tumor tissue	Adjacent tissue	
Total number of patients enrolled	40	20	
Age at first diagnosis (years)	49.90 ± 10.20	48.30 ± 8.68	0.000
Ethnicity			0.566
Han	25 (62.5)	14 (70.0)	
Others	15 (37.5)	6 (30.0)	
Tumor size (cm)			0.756
≤ 4	30 (75.0)	16 (80.0)	
> 4	10 (25.0)	4 (20.0)	
Differentiation			1.000
Poor	10 (25.0)	5 (25.0)	
Middle/high	30 (75.0)	15 (75.0)	
FIGO stage			0.378
IA	4 (10.0)	0 (0.0)	
IB	15 (37.5)	8 (40.0)	
IIA	20 (50.0)	11 (55.0)	
IIB	1 (2.5)	1 (5.0)	

### Abnormal expression of CXC chemokines in CC patients

**Fig. 1:**
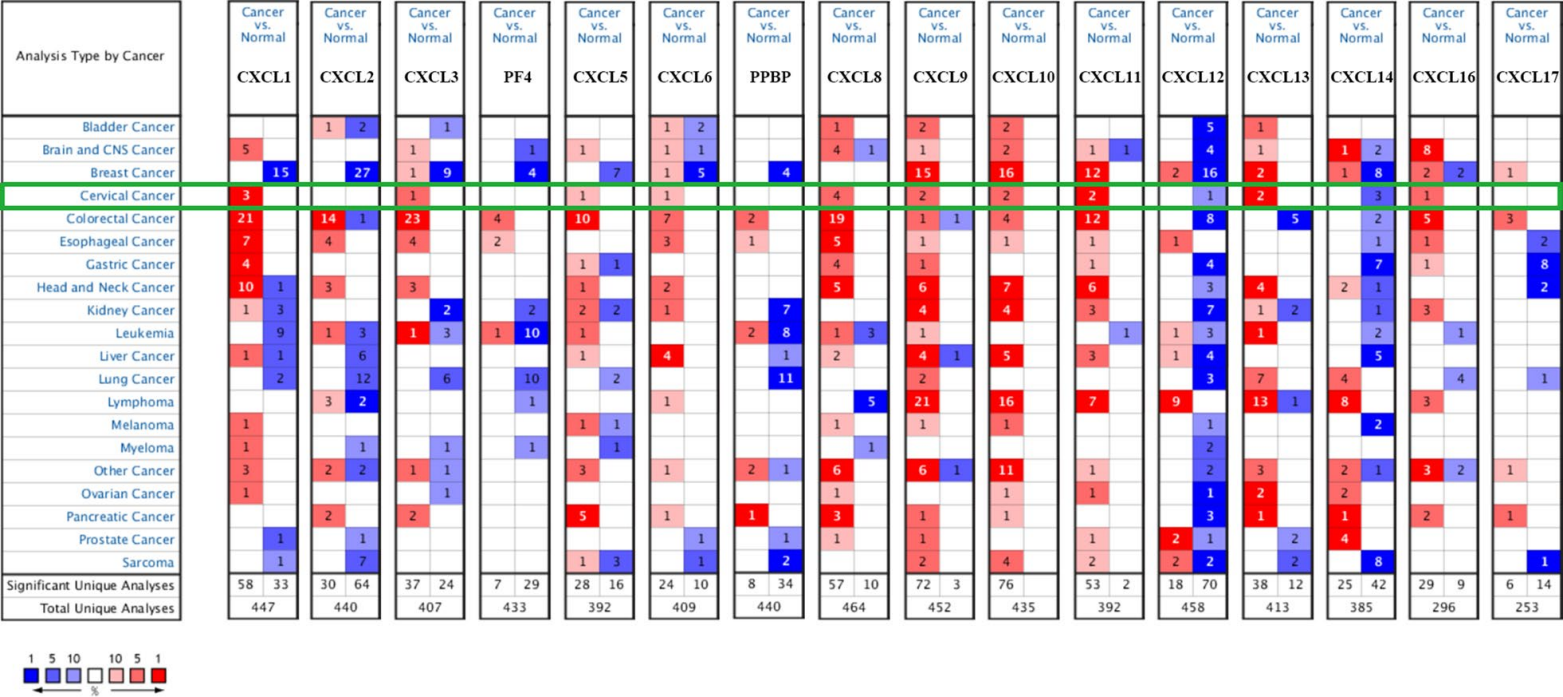
mRNA levels of CXC chemokines in diverse types of cancers (ONCOMINE). It shows the numbers of datasets with statistically significant mRNA over-expression (red) or down-regulated expression (blue) of CXC chemokines by Students’ *t*-test. The parameters are as follows, *p*-value: 0.01, fold change: 2.0, gene rank: 10%

**Fig. 2:**
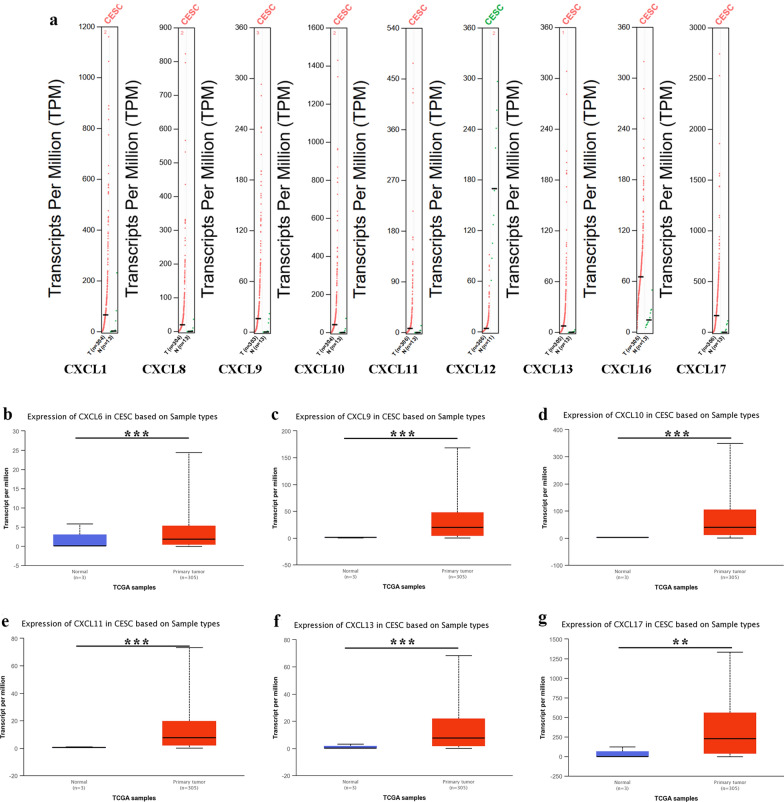
The expression of distinct CXC chemokines in cervical cancer tissues and normal or adjacent cervical tissues. **a** The red color represents high expression, while the green color shows low expression in cervical tumor tissues in the GEPIA database. **b**–**g** The blue color represents normal or adjacent cervical tissues, red color represents cervical cancer tissue in the UALCAN database. **p < 0.01, ***p < 0.001

To investigate the transcription level of CXC chemokines between tumor and adjacent or normal tissues in CC, we performed an analysis using the ONCOMINE, GEPIA and UALCAN database. Sixteen CXC chemokines were retrieved using the ONCOMINE databases. The results are presented in Fig. 1 and Table 2. The transcriptional level of CXCL1/3/5/6/8/9/10/11/13/16 in cervical cancer tissues was significantly elevated, while the transcriptional level of CXCL12/14 was significantly lower than that of adjacent cancer tissue. In Zhai cervix statistics [27], CXCL1/3/5/6/8/13 were overexpressed in cervical tumor tissues versus tumor-adjacent tissues. In Scotto Cervix 2 Statistics [28], CXCL1/8/9/10/11/13 were elevated in CC tissues relative to the adjacent tissues of the tumor. Similarly, CXCL8/9/10/11 were overexpressed in CC tissues instead of para-carcinoma tissue in Biewenga Cervix Statistics [29]. In the GEPIA database, the results indicated that the expressional levels of CXCL1/8/9/10/11/13/16/17 were increased in tumor tissues rather than normal tissues, while CXCL12 was reduced (Fig. 2a). In the UALCAN database, as expected, the transcriptional levels of CXCL6 (*p* = 1.18e−4), CXCL9 (*p* = 1.63e−12), CXCL10 (*p* = 7.77e−16), CXCL11 (*p* = 8.36e−13), CXCL13 (*p* = 2.81e−7) and CXCL17 (*p* = 1.74e−3) in cervical tissues were significantly elevated (Fig. 2b–g). Since the normal control of the study in the ONCOMINE database came from para-carcinoma tissues rather than normal tissues, it was rational to acquire diverse results from the three databases. In addition, the sample size was not sufficient to capture variability as there were only three normal cervical patients in the UALCAN database. This part of the data has yet to be further confirmed in clinical practice. Taken together, these data suggest that these CXC chemokines play a significant role in the tumorigenesis and progression of cervical cancer.

**Table 2:** The significant changes of CXC chemokines expression in transcription level between different types of CC

TLR	Type	Fold change	*p*-value	*t*-test	References
CXCL1	High-grade cervical squamous intraepithelial neoplasia epithelia vs. normal	7.048	2.83e−5	6.446	Zhai Cervix Statistics [27]
	Cervical squamous cell carcinoma epithelia vs. normal	4.462	1.33e−6	5.866	Zhai Cervix Statistics [27]
	Cervical squamous cell carcinoma vs. normal	3.895	5.62e−5	4.166	Scotto Cervix 2 Statistics [28]
CXCL3	High-grade cervical squamous intraepithelial neoplasia epithelia vs. normal	2.797	6.20e−4	4.111	Zhai Cervix Statistics [27]
CXCL5	High-grade cervical squamous intraepithelial neoplasia epithelia vs. normal	5.994	0.002	4.393	Zhai Cervix Statistics [27]
CXCL6	High-grade cervical squamous intraepithelial neoplasia epithelia vs. normal	2.976	0.003	3.969	Zhai Cervix Statistics [27]
CXCL8	Cervical squamous cell carcinoma vs. normal	13.807	5.04e−9	6.765	Scotto Cervix 2 Statistics [28]
	Cervical squamous cell carcinoma vs. normal	3.974	8.10e−8	7.474	Biewenga Cervix Statistics [29]
	Cervical squamous cell carcinoma epithelia vs. normal	3.789	8.81e−5	4.318	Zhai Cervix Statistics [27]
CXCL9	Cervical squamous cell carcinoma vs. normal	8.529	8.45e−9	11.614	Biewenga Cervix Statistics [29]
	Cervical squamous cell carcinoma vs. normal	3.523	1.47e−6	5.246	Scotto Cervix 2 Statistics [28]
CXCL10	Cervical squamous cell carcinoma vs. normal	3.982	4.89e−11	11.782	Biewenga Cervix Statistics [29]
	Cervical squamous cell carcinoma vs. normal	3.050	1.15e−4	3.947	Scotto Cervix 2 Statistics [28]
CXCL11	Cervical squamous cell carcinoma vs. normal	3.700	4.13e−16	12.455	Biewenga Cervix Statistics [29]
	Cervical squamous cell carcinoma vs. normal	3.555	2.09e−5	4.476	Scotto Cervix 2 Statistics [28]
CXCL13	Cervical squamous cell carcinoma vs. normal	19.655	9.32e−11	7.981	Scotto Cervix 2 Statistics [28]
	Cervical squamous cell carcinoma epithelia vs. normal	5.835	3.49e−5	4.636	Zhai Cervix Statistics [27]

**Fig. 3:**
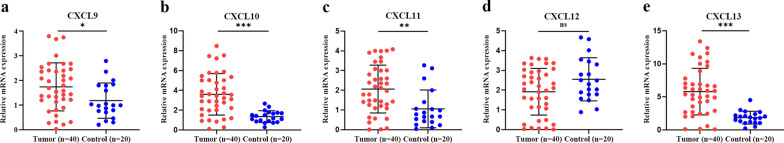
Expression of CXC chemokines between cervical cancer tissues and adjacent tissues were analyzed by qRT-PCR by Student's *t*-test or Mann–Whitney *U* test. CXCL9/10/11/13 were significantly up-regulated in cervical cancer tissues compared with adjacent tissues. **a** CXCL9. **b** CXCL10. **c** CXCL11. **d** CXCL12. **e** CXCL13.*p < 0.05, **p < 0.01, ***p < 0.001

**Fig. 4:**
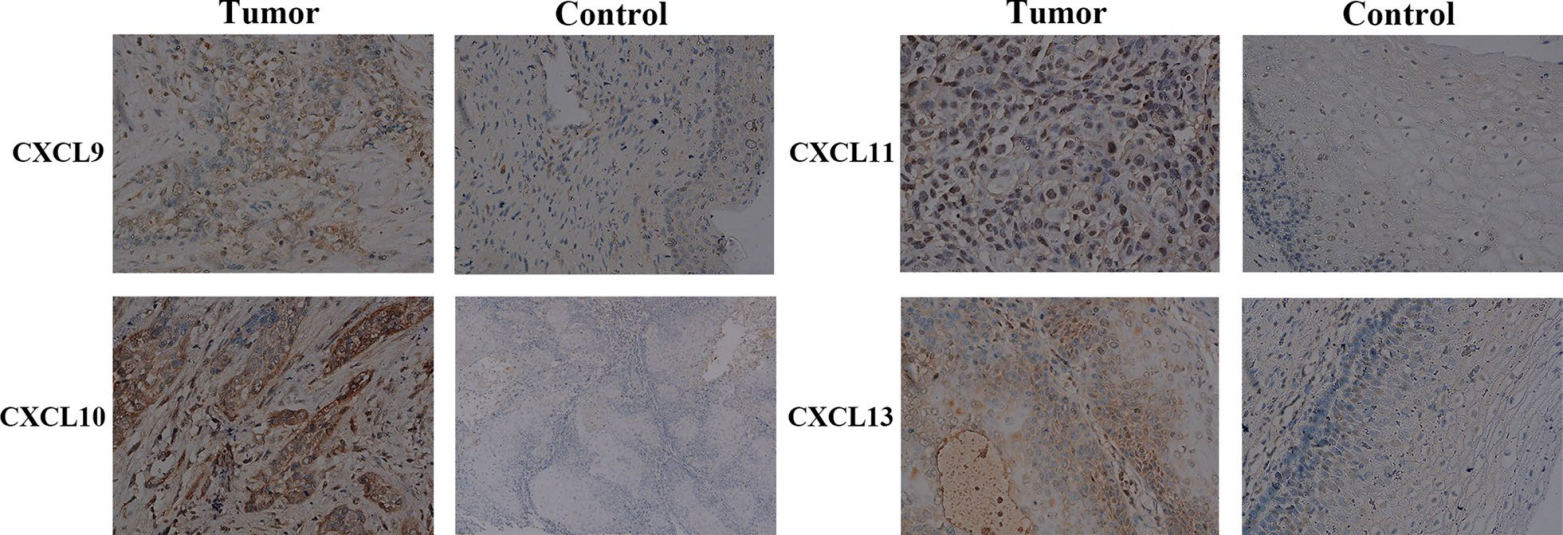
Representative immunohistochemical images of distinct CXC chemokines in cervical cancer tissues and adjacent cervical tissues (χ400). We can observe that CXCL9/10/11/13 was highly expressed in cervical cancer tissues compared to adjacent cervical tissues

The mRNA expression of CXCL9/10/11/12/13 in cervical cancer tissues and adjacent cancer tissues was also examined by qRT-PCR (Fig. 3). We verified CXCL9/10/11/13 because these factors were expressed at the intersection of the three databases. CXCL12 was detected owing to it was the only one with a tendency of low expression in the two databases. CXCL9 (*p* = 0.027), CXCL10 (*p* < 0.001), CXCL11 (*p* = 0.002) and CXCL13 (*p* < 0.001) were significantly up-regulated in tumor tissues compared with tumor-adjacent tissues, while the expression of CXCL12 (*p* = 0.149) was down-regulated. We further performed IHC on CXCL9/10/11/13 to detect their protein expression in cervical cancer tissues and adjacent tissues. We found that the protein expression CXCL9 (*p* = 0.012), CXCL10 (*p* = 0.003), CXCL11 (*p* = 0.011), CXCL13 (*p* = 0.004) were higher in tumor tissues than that in adjacent tissues (Fig. 4). These were consistent with the findings in the databases and also further verified the transcriptional expression of CXCL9/10/11/13 in cervical cancer.

### The prognostic value of CXC chemokines in cervical cancer

**Fig. 5:**
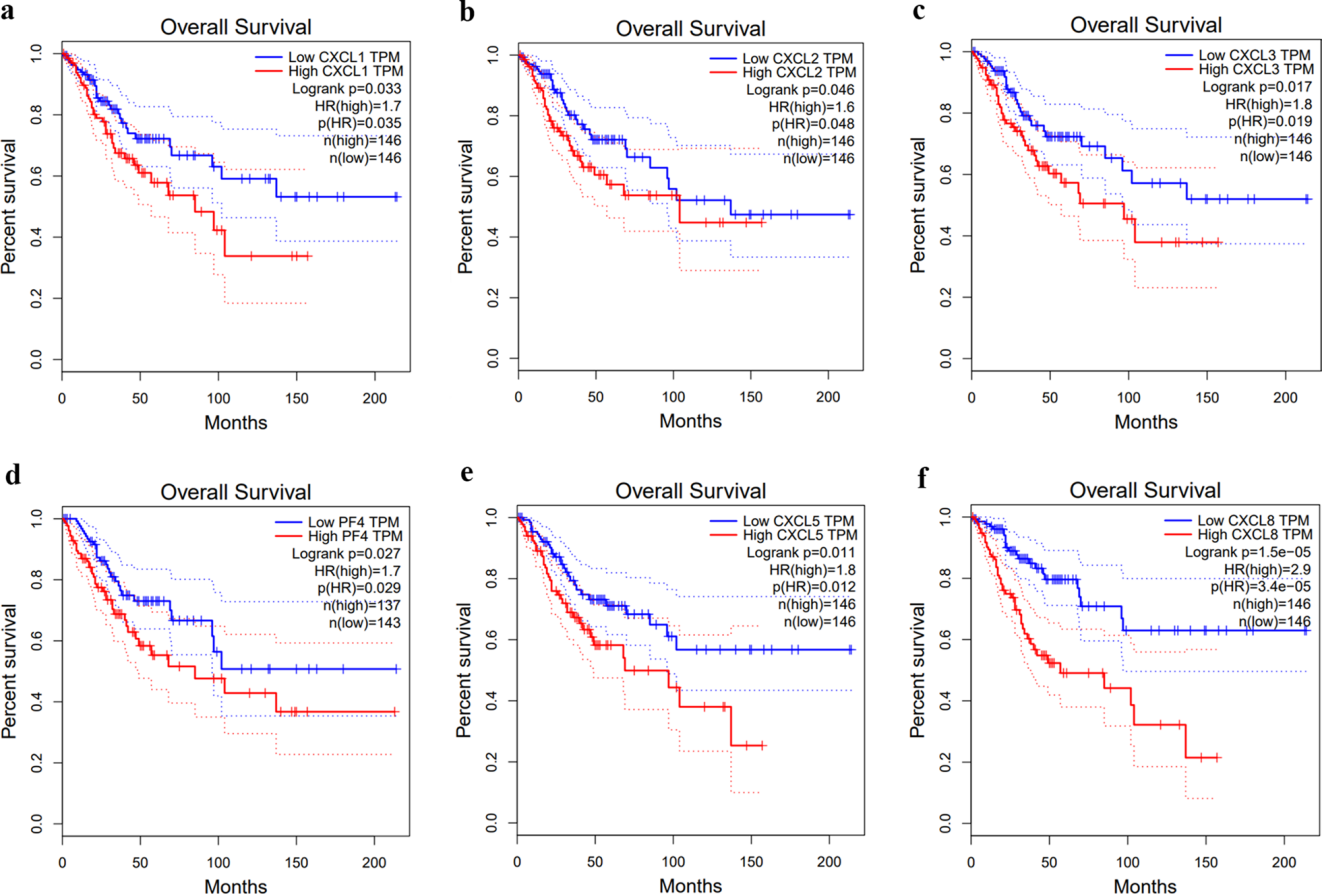
The prognostic value of different expressed CXC chemokines in the overall survival curve of patients with cervical cancer (GEPIA). **a** CXCL1. **b** CXCL2. **c** CXCL3. **d** CXCL4. **e** CXCL5. **f** CXCL8

We conducted an analysis in the GEPIA database to explore the value of differentially expressed CXC chemokines in the progression of CC by evaluating the correlation between differentially expressed CXC chemokines and clinical outcomes. The low transcriptional levels of CXCL1 (*p* = 0.033), CXCL2 (*p* = 0.046), CXCL3 (*p* = 0.017), CXCL4 (*p* = 0.027), CXCL5 (*p* = 0.011) and CXCL8 (*p* = 1.5e−5) were significantly associated with longer OS of CC patients (Fig. 5).

### Genetic alteration of CXC chemokine in cervical cancer

**Fig. 6:**
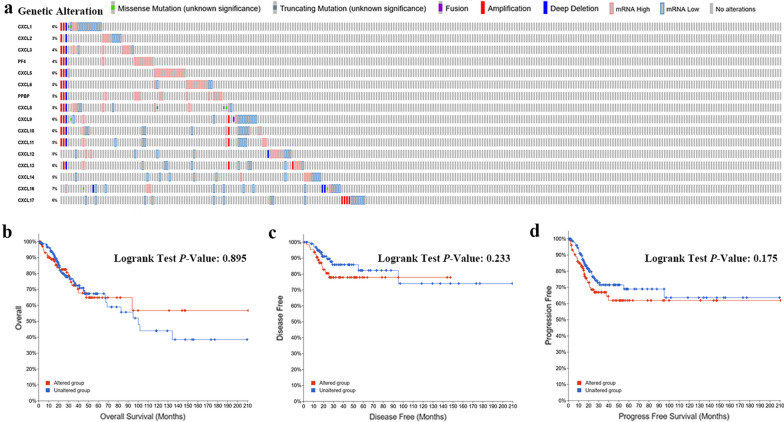
Analysis of genetic alteration of CXC chemokine in cervical cancer. **a** CXC chemokines’ mutation analysis in cervical cancer (cBioPortal). **b** The overall survival curve analysis of the altered group and unaltered group (*p* = 0.895). **c** The disease-free survival curve analysis of the altered group and unaltered group (*p* = 0.233). **d** The progress-free survival curve analysis of the altered group and unaltered group (*p* = 0.175)

We analyzed the genetic alterations of differentially expressed CXC chemokines by using the TCGA dataset in the cBioPortal. We found that a high mutation rate of CXC chemokines (42%) was observed in CC patients. Among 293 cervical cancer patients, the genetic alteration was found in 124 cervical cancer patients. As a result, CXCL1, CXCL2, CXCL3, CXCL4, CXCL5, CXCL6, CXCL7, CXCL8, CXCL9, CXCL10, CXCL11, CXCL12, CXCL13, CXCL14, CXCL6 and CXCL17 were altered in 6%, 3%, 4%, 4%, 6%, 5%, 5%, 5%, 6%, 6%, 5%, 5%, 6%, 5%, 7% and 6% of the cervical cancer samples, respectively (Fig. 6a). In addition, we compared the overall survival, disease-free survival and progress-free survival between the CXC chemokine mutant group and the non-mutant group. Unfortunately, there were no difference in overall survival (*p* = 0.895), disease-free survival (*p* = 0.233) and progress-free survival (*p* = 0.175) between the CXC chemokine mutant group and the non-mutant group (Fig. 6b, c).

### Analysis of adjacent gene network and interaction of CXC chemokines in cervical cancer

**Fig. 7:**
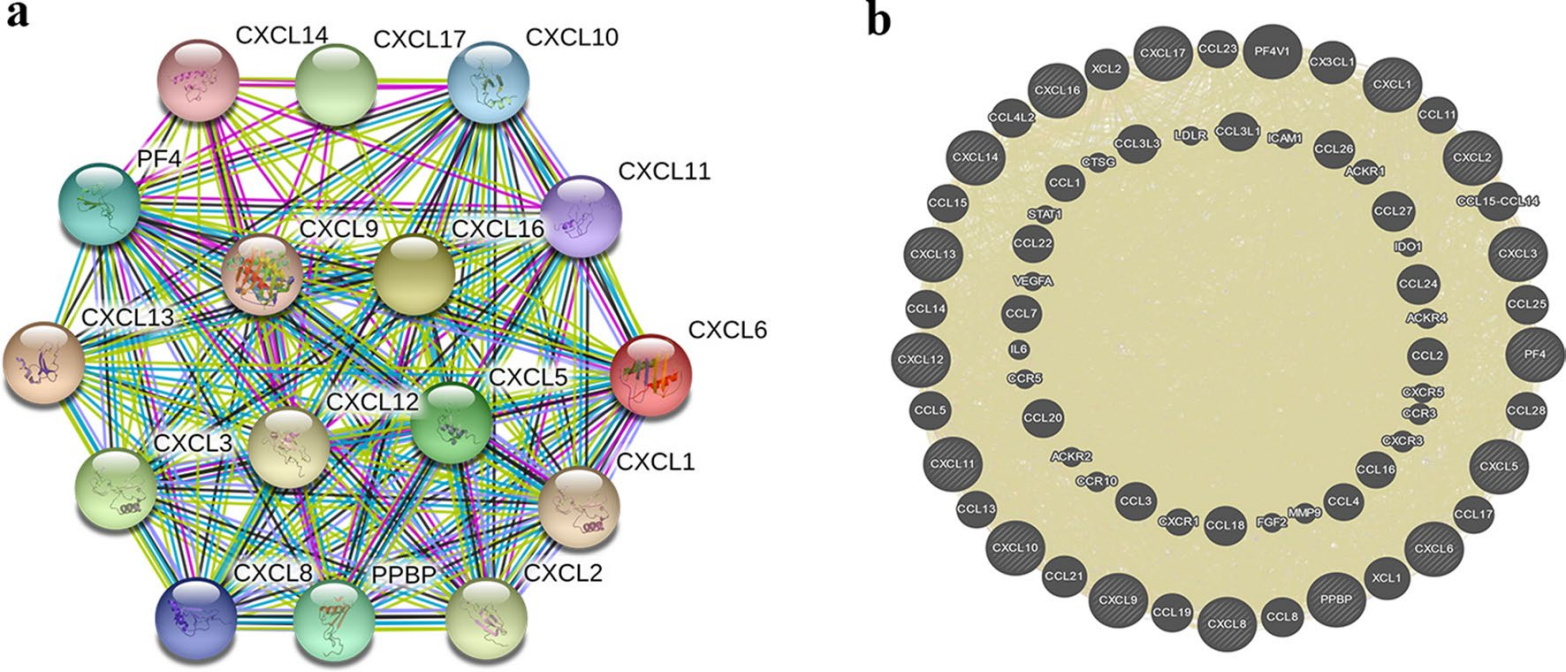
Protein–protein interaction network of CXC chemokines. **a** STRING database**. b** GeneMANIA database

To further explore the potential interactions between differentially expressed CXC chemokines and adjacent genes, we performed a PPI network analysis in STRING (Fig. 7a). The results revealed that the function of the differentially expressed CXC chemokines was related to chemokine signaling pathways and inflammation response. Results on the GeneMANIA website also showed the differential expressed CXC chemokines’ function was primarily connected with cell chemotaxis, chemokine receptor binding, and chemokine activity (Fig. 7b).

### Predicting the function and pathway of CXC chemokines in patients with cervical cancer

**Fig. 8:**
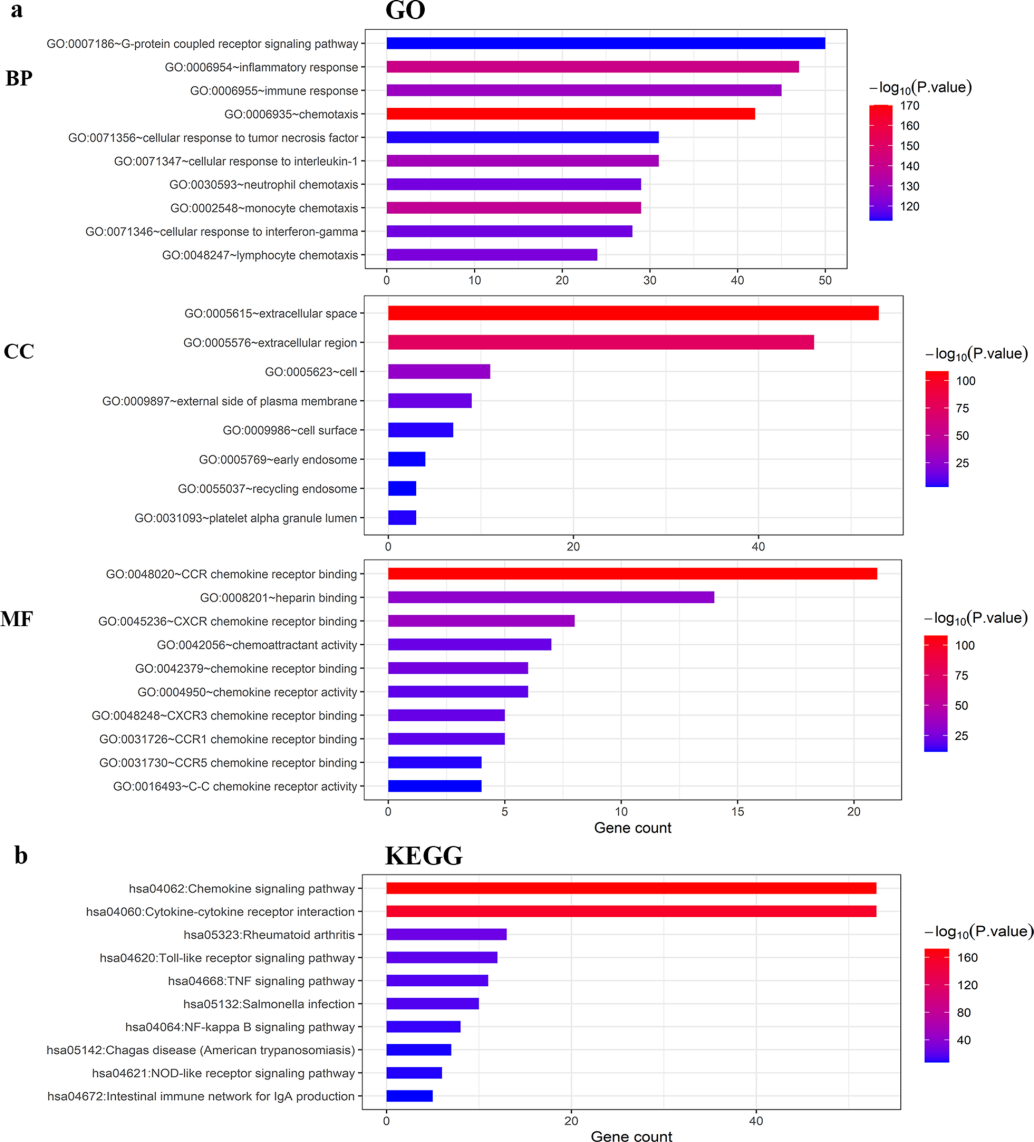
GO and KEGG enrichment analysis of CXC chemokines (DAVID). **a** Bar-plot of GO enrichment in biological process terms, cellular component terms and molecular function terms, respectively. **b** Bar-plot of KEGG enrichment terms

The function and pathway of the differentially expressed CXC chemokines and their neighboring genes were investigated using DAVID 6.8. As shown in Fig. 8a, GO term analysis showed that the differentially expressed CXC chemokines were mainly located in the extracellular space, extracellular region, cell, external side of plasma membrane and cell surface, where they participated in chemotaxis, inflammatory response, monocyte chemotaxis, cellular response to interleukin-1 and immune response. They acted as CCR chemokine receptor binding, CXCR chemokine receptor binding, heparin binding, chemokine receptor binding and chemoattractant activity. KEGG pathway analysis showed enrichment of chemokine signaling pathway, cytokine–cytokine receptor interaction, rheumatoid arthritis, Toll-like receptor (TLR) signaling pathway, TNF signaling pathway (Fig. 8b).

### Analysis of kinase and transcription factor targets in patients with cervical cancer

The TRRUST and Linkedomics databases were used to explore the possible kinase and TF targets of differential CXC chemokines in the cervical cancer microenvironment. As shown in Table 3, RELA and NFKB1 were the key TFs for CXCL1/2/5/8/10/12 (*p* = 1.09e−07 and *p* = 1.14e−07, respectively), SP1 was the key TF for CXCL1/5/14 (*p* = 0.00683) in the TRRUST database. Moreover, the top two kinase targets of CXC chemokines were studied from the LinkedOmics database (Table 4). Src family tyrosine kinases (LYN and LCK) were the primary kinase target of CXCL10/11/16. The kinase target of CXCL5 was the PAK family kinases (PAK2 and PAK3).

**Table 3:** Key regulated factor of CXC chemokines in CC (TRRUST)

Key TF	Description	Regulated gene	*p*-value	FDR
RELA	v-rel reticuloendotheliosis viral oncogene homolog A (avian)	CXCL1, CXCL2, CXCL5, CXCL8, CXCL10, CXCL12	1.09e−07	1.71e−07
NFKB1	Nuclear factor of kappa light polypeptide gene enhancer in B-cells 1	CXCL1, CXCL2, CXCL5, CXCL8, CXCL10, CXCL12	1.14e−07	1.71e−07
SP1	Sp1 transcription factor	CXCL1, CXCL5, CXCL14	0.00683	0.00683

**Table 4:** The kinase target networks of CXC chemokines in CC (LinkedOmics)

CXC chemokines	Enriched kinase target	Description	Leading EdgeNum	*p*-value
CXCL1	Kinase_ATR	ATR serine/threonine kinase	16	0
	Kinase_CHUK	Conserved helix–loop–helix ubiquitous kinase	10	0
CXCL2	Kinase_RPS6KB1	Ribosomal protein S6 kinase B1	6	0
	Kinase_ATM	ATM serine/threonine kinase	38	0
CXCL3	Kinase_GRK6	G-protein-coupled receptor kinase 6	2	0
	Kinase_ATM	ATM serine/threonine kinase	37	0
CXCL5	Kinase_PAK2	p21 (RAC1) activated kinase 2	7	0
	Kinase_PAK3	p21 (RAC1) activated kinase 3	3	0
CXCL6	Kinase_DAPK1	Death-associated protein kinase 1	6	0
	Kinase_EGFR	Epidermal growth factor receptor	20	0
CXCL9	Kinase_ZAP70	Zeta chain of T-cell receptor associated protein kinase 70	4	0
	Kinase_LCK	LCK proto-oncogene, Src family tyrosine kinase	23	0
CXCL10	Kinase_LYN	LYN proto-oncogene, Src family tyrosine kinase	21	0
	Kinase_LCK	LCK proto-oncogene, Src family tyrosine kinase	21	0
CXCL11	Kinase_LCK	LCK proto-oncogene, Src family tyrosine kinase	22	0
	Kinase_LYN	LYN proto-oncogene, Src family tyrosine kinase	21	0
CXCL12	Kinase_RPS6KA4	Ribosomal protein S6 kinase A4	11	0
	Kinase_PLK3	Polo-like kinase 3	6	0
CXCL13	Kinase_LCK	LCK proto-oncogene, Src family tyrosine kinase	22	0
	Kinase_SYK	Spleen-associated tyrosine kinase	15	0
CXCL14	Kinase_CDK1	Cyclin-dependent kinase 1	74	0
	Kinase_MAP3K8	Mitogen-activated protein kinase kinase kinase 8	7	0
CXCL16	Kinase_LCK	LCK proto-oncogene, Src family tyrosine kinase	23	0
	Kinase_LYN	LYN proto-oncogene, Src family tyrosine kinase	22	0
CXCL17	Kinase_ADRBK1	G-protein-coupled receptor kinase 2	8	0
	Kinase_IGF1R	Insulin-like growth factor 1 receptor	7	0

### CXC chemokines and immune cell infiltration

**Fig. 9:**
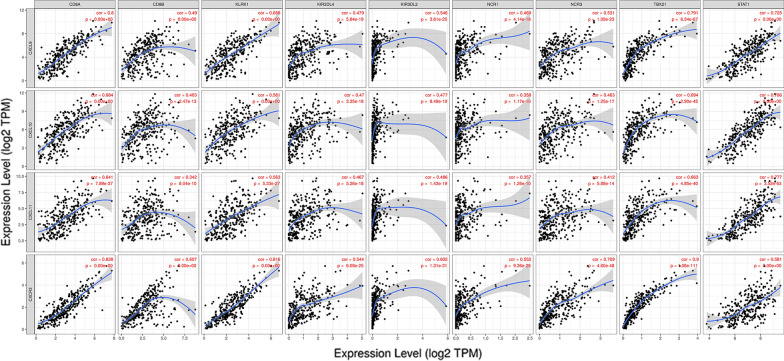
The correlation study of CXCL9–11, CXCR3 and CD8+ T cells, natural killer (NK) cells, T helper 1 (Th1) cells in cervical cancer (TIMER). The mRNA expression levels of CXCL9–11, CXCR3 are related to CD8+ T cells, NK cells, and Th1 cells. Molecular markers of CD8+ T cells: CD8A and CD8B, NK cells: KLRK1, KIR2DL4, KIR3DL2, NCR1 and NCR3, and Th1 cells: TBX21 and STAT1

As a component of the tumor microenvironment, CXC chemokines were closely knitted with immune cells. Since the experimental validation was performed only on CXCL9/10/11/13, the current work primarily focused on the relationship between CXCL9/10/11 and their corresponding receptor CXCR3 in the TIMER database. The expression of CXCL9/10/11 and CXCR3 was negatively correlated with tumor purity, indicating that they were highly expressed in infiltrating immune cells rather than tumor cells in TME. The expression of CXCL9/10/11 was positively associated with the infiltration of CD8+ T cell, CD4+ T cell, neutrophils and dendritic cells (all *p* < 0.05, Additional file 1: Figure S1a–c). Moreover, there was a positive correlation between the expression of CXCR3 and the infiltration of B cell, CD8+ T cell, CD4+ T cell, macrophages, neutrophils and dendritic cells (all *p* < 0.05, Additional file 1: Figure S1d). It is universally accepted that CXCR3 is commonly expressed in CD8+ T cells, NK cells, and Th1 cells. We further evaluated the molecular markers of CXCL9/10/11, CXCR3 and CD8+ T cells, NK cells and Th1 cells. We found that the expression of CXCL9/10/11 and CXCR3 was positively correlated with these cell markers (Fig. 9).

## Discussion

Although there have been some improvements in the early detection and treatment of cervical cancer, it is still the leading cause of cancer-related deaths among females. CXC chemokines, as a component of TME, affect the occurrence and development of cancer. In our study, we comprehensively analyzed the prognostic value and biological function of CXC chemokines in CC using public databases. We found that the expression of CXCL1/3/5/6/8/9/10/11/13/16/17 was up-regulated in tumor tissues, while the expression of CXCL12/14 was down-regulated. The OS of CC patients with low transcription levels of CXCL1/2/3/4/5/8 was longer. In addition, we noticed that the CCL family was related to CXC chemokines neighboring alteration. The enrichment analysis of CXC chemokines was mainly connected with the chemokine signaling pathway, etc., RELA, NFKB1, LCK and PAK2 were the key TF and kinase targets of CXC chemokines, respectively. In short, these data indicate that differentially expressed CXC chemokines may play an important role in CC.

We have not reached a consistent conclusion on the expression of CXC chemokines in the three public databases. Considering that the normal control of this study in the ONCOMINE database was from cancer adjacent tissues rather than normal tissues, and there were only three normal cervical samples in the UALCAN database that were not sufficient to capture variability. Therefore, it was rational to obtain different results from the three databases. In addition, as for qRT-PCR analysis, the expression of CXCL9/10/11/12/13 was consistent with the data in the database. Unfortunately, there was no statistically significant difference in the expression of CXCL12 between tumor and para-cancerous tissues. One possible interpretation is that cervical expression of CXCL12 is heterogeneous, tending to be inhibited as the tumor grows [30].

The occurrence and development of cervical cancer is a complex and multi-faceted process. We investigated the molecular characteristics of CXC chemokines that were significantly differentially expressed in CC. The differentially expressed CXC chemokines in CC had frequent genetic alterations with a mutation rate of 42%, of which increased mRNA expression was the most alteration. The CCL family was associated with CXC chemokines neighboring alteration, including CCL1, CCL2, CCL8 and CCL21, etc. There were different but similar functions performed by these CCL factors in the tumor. It has been reported that CCL1, CCL2 and CCL21 affect tumor metastasis and progression through direct or indirect interactions with lymph nodes [31–33]. CCL8 was an independent prognostic factor for patients with CC, and increased secretion of CCL8 promoted tumor progression [34, 35]. These results revealed that CXC chemokines may inhibit or promote tumor growth by influencing the alterations of neighboring genes in the tumor microenvironment.

Then via GO enrichment analysis and KEGG pathway enrichment analysis, we found that the functions of these genes were primarily related to the chemokine signaling pathway, cytokine–cytokine receptor interaction, and the Toll-like receptor signaling pathway. Previous studies have shown that chemokine signaling pathways affect tumor proliferation, angiogenesis and metastasis [8, 36]. For example, the increase of CXCL1/2/8 level regulated by AKIP 1 played an important role in the angiogenesis and growth of cervical cancer [37]. However, CXCL9/10/11, as anti-angiogenic chemokines, inhibited the angiogenesis of cervical cancer through its receptor CXCR3-B [8]. CXCL13 and CXCR5 have also been revealed in some cancers. The hypermethylation of a single CpG dinucleotide in the promoter region of the CXCL13 gene promoted the migration of cervical cancer cells [38]. Silencing of CXCL12 altered the homeostatic autocrine and paracrine CXCR4 signaling to endocrine communication in the tumor microenvironment, leading to tumor progression and metastases [30]. In addition, TLRs are I intermembrane proteins, and TLR signaling pathways in tumor cells have been shown to influence cancer progression. Stimulating TLRs in tumor cells fostered chronic inflammation that drove cancer cell proliferation, migration and angiogenesis, and established a tumor microenvironment that impaired the immune system and facilitated tumor growth [39, 40]. These data indicated that the differentially expressed CXC chemokines in CC were potential therapeutic targets.

By analyzing the differential expression of CXC chemokines in cervical cancer, we identified three key transcription factors (RELA, NFKB1 and SP1). RELA phosphorylation resulted in cancer progression by regulating NF-κB signaling [41]. Some studies have revealed an association between elevated RELA expression and poor survival in cancer [42]. Moreover, NFKB1 was a tumor suppressor by inhibiting cell proliferation, colony formation and migration in cervical cancer, while the mutation could weaken the tumor-suppressing function of NFKB1 [43, 44]. Sp1 may lead to radioresistance by targeting CDK1 to inhibit G2/M phase block, thereby affecting the prognosis of patients [45]. In addition, our data suggested that LCK, LYN, PAK2 and PAK3 may be kinase targets for differential expression of CXC chemokines. It was related to Src family tyrosine kinases and PAK family kinases. These kinases were involved in the occurrence and development of tumors by regulating the proliferation, migration, invasion and apoptosis of tumor cells [46–48]. All in all, differentially expressed CXC chemokines may affect tumor development and progression by regulating these transcription factors and kinases in cervical cancer.

In the TME of cervical cancer, CXC chemokines could control the migration and localization of immune cells, and immune cells could be a factor of immunotherapy and clinical outcome by affecting tumor development and progression [49, 50]. It was well known that CD8+ T cells and NK cells can be recruited via the CXCL9–11/CXCR3 axis, resulting in tumor-suppressing with a form of paracrine [51]. In our study, CXCL9/10/11 and their ligand CXCR3 were positively related to CD8+ T cells and NK cells. The results suggested that CXCL9/10/11 in the tumor microenvironment may recruit CD8+ T cells and NK cells through the CXCL9–11/CXCR3 axis, thereby improving the survival of cervical cancer patients by inhibiting the development and progression of tumors. Drugs that augmented paracrine CXCL9/10/11 expression and deactivated CXCR3 expression on tumor cells, have shown anti-tumor activity in several tumor models. At present, the treatment of anti-CXCR 3 is promising. CXCR3 antagonist has been shown to inhibit the implantation and growth of tumor cells in vitro, and inhibit lung metastasis in a vivo model [52]. However, it is still worth exploring in cervical cancer. In short, these results indicated that CXC9/10/11 could be regarded as anti-tumor target by attracting immune cells.

In our study, experiments and several online bioinformatic platforms have systematically analyzed the expression, mutations, and related pathway value of CXC chemokines in CC. We look forward to paying more attention to the therapeutic effect of CXC chemokines in cervical cancer in future research. Through a more profound and comprehensive exploration of the key transcription factors, kinase targets and immune cells, the mechanism of CXC chemokines in the tumor microenvironment can be better revealed. There were some limitations in our study. First of all, due to the limitation of the online database, there were only three normal cervical patients in the UALCAN database, which was insufficient to capture variability. Therefore, clinical studies are needed to verify the conclusions. Second, analysis on the transcriptional and translational level can certainly reflect some aspects of immune status, but not global changes. Third, we should collect more clinical specimen data for experimental verification. Since this study only verified the mRNA and protein expression of some CXC chemokines, more indicators are supposed to be further verified in the experiment.

## Conclusions

In conclusion, we performed a comprehensive analysis of CXC chemokines via clinical data and some online tumor databases. Patients with low transcriptional levels of CXCL1/2/3/4/5/8 were significantly associated with better prognosis, suggesting a role in tumorigenesis and development. CXCL9–11 were highly correlated with CD8+ T cells, NK cells, and Th1 cells in the tumor microenvironment, indicating that they can play a certain role in the anti-tumor immune response. In addition, we found that RELA, NFKB1 and SP1 were the key transcription factors of CXC chemokines, and LCK, LYN, PAK2, and PAK3 were the kinase targets of CXC chemokines. These results suggested that the interaction between these factors and CXC chemokines was involved in the mechanism of progression in CC. We hope that the differentially expressed CXC chemokine data could provide ideas and insights on the selection of prognostic markers and immunotherapy for cervical cancer.

## Supplementary Information

**Additional file 1: Figure S1. **Correlation between the expression of CXCL9–11, CXCR3 and immune cells. Immune cells include B cell, CD8+T cell, CD4+T cell, macrophage, neutrophil and dendritic cell. **a** CXCL9. **b** CXCL10. **c** CXCL11. **d** CXCR3.

## Data Availability

The datasets supporting the conclusions of this article are included within the article.

## Abbreviations

TME: Tumor microenvironment
CC: Cervical cancer
qRT-PCR: Quantitative real-time PCR
IHC: Immunohistochemistry
OS: Overall survival
TCGA: The Cancer Genome Atlas
PPI: Protein–protein interaction
GO: Gene Ontology
KEGG: Kyoto Encyclopedia of Genes and Genomes
BP: Biological processes
CC: Cellular components
MF: Molecular function
TF: Transcription factor
GSEA: Gene Set Enrichment Analysis
NK: Natural killer
Th1: T helper 1
TLR: Toll-like receptor
