# Supplementary figures and images for: Analysis of therapeutic targets and prognostic biomarkers of CXC chemokines in cervical cancer microenvironment
Source: Cancer Cell Int. 2021 Jul 28;21:399. doi: 10.1186/s12935-021-02101-9 (PMC8317415; doi:10.1186/s12935-021-02101-9)

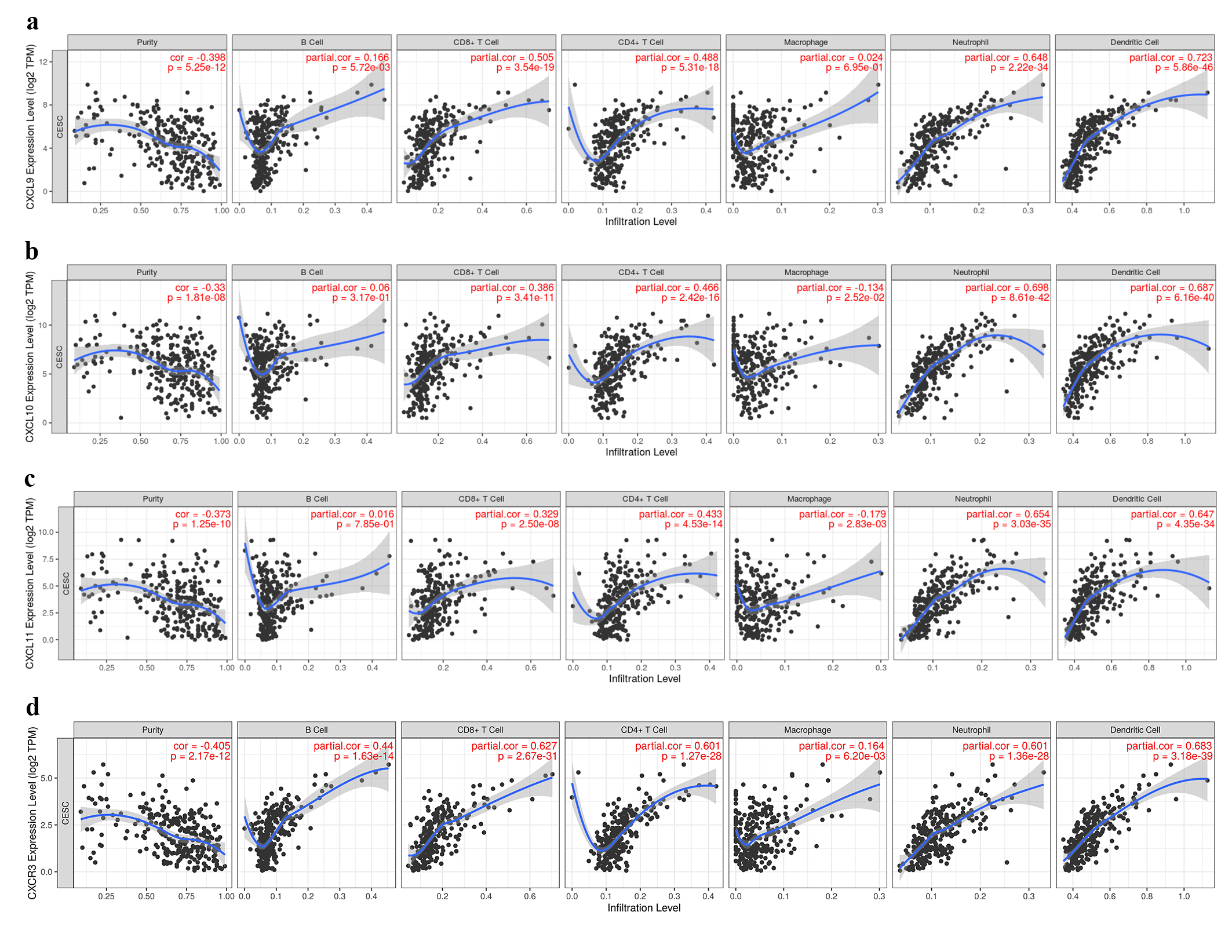

Supplement: Supplementary file 1 — Additional file 1: Figure S1. Correlation between the expression of CXCL9–11, CXCR3 and immune cells. Immune cells include B cell, CD8+T cell, CD4+T cell, macrophage, neutrophil and dendritic cell. a CXCL9. b CXCL10. c CXCL11. d CXCR3. [file 12935_2021_2101_MOESM1_ESM.tif]
